# ASO Author Reflections: Age, Histology, and Treatment with Surgery are Associated with Increased 5-Year Cause-Specific Survival Despite Relative Undertreatment Among Patients with Primary Tracheal Cancers

**DOI:** 10.1245/s10434-024-16574-1

**Published:** 2024-11-20

**Authors:** Michael Larkins

**Affiliations:** 1 Brody School of Medicine, East Carolina University, Greenville, USA; 2 Department of Emergency Medicine, Boonshoft School of Medicine, Wright State University, Dayton, OH USA

## Past

Primary tracheal cancers (PTCs) are rare neoplasms, with an estimated incidence of just one per one million individuals.^1^ Current literature recommends surgical management of these cancers, though there is a dearth of literature on general outcomes and a relative undertreatment of PTC with surgery has been reported.^2^ Even less literature exists exploring the role of other oncologic treatment modalities and the role of multimodal therapy, and currently no consensus guidelines exist for the management of these cancers.

## Present

We performed a population-level analysis of patients with PTC identified via the Surveillance, Epidemiology, and End Results (SEER) database to assess for factors associated with increased 5-year cause-specific survival (CSS).^3^ On multivariate analysis, patient age < 65 years at diagnosis (hazard ratio (HR) = 0.64, *p* < 0.001), non-squamous cell carcinoma (SCC) histologies (namely, adenoid cystic carcinoma (ACC); HR = 0.22, *p* < 0.001), and treatment with surgery (HR = 0.43, *p* < 0.001) were all associated with increased CSS. Despite these findings, 55% of the entire cohort (*n* = 380) did not receive surgical treatment, with most of these patients having SCC histology (58%).

## Future

These results suggest that significant differences in outcomes with respect to patient age and PTC histology exist among the US population. Furthermore, while surgery was found to improve survival among patients with PTC, a relative undertreatment trend continues to exist, especially among those with SCC of the trachea. More research is needed to assess these trends and to serve as the basis for future evidence-based guideline creation.
