# Correction: ASO Author Reflections: Age, Histology, and Treatment with Surgery are Associated with Increased 5-Year Cause-Specific Survival Despite Relative Undertreatment Among Patients with Primary Tracheal Cancers

**DOI:** 10.1245/s10434-025-18193-w

**Published:** 2025-08-18

**Authors:** Michael Larkins

**Affiliations:** 1 Brody School of Medicine, East Carolina University, Greenville, USA; 2 Department of Emergency Medicine, Boonshoft School of Medicine, Wright State University, Dayton, OH USA

**Correction to: Annals of Surgical Oncology (2024) 32:880–881** 10.1245/s10434-024-16574-1

The following disclaimer was missing from the original online version of this article. The original article was corrected:

The views and opinions expressed in this article/presentation are those of the author and do not necessarily reflect official policy or position of the United States Air Force, Defense Health Agency, Department of Defense, or U.S. Government.

